# Harnessing Digital Innovation for Diabetes Care: Insights From the Action4Diabetes–CorrelAid Data4Good Collaboration

**DOI:** 10.2196/89357

**Published:** 2026-04-17

**Authors:** Sze May Ng, Michael Aydinbas, Tyla Martin, Darren Jia Chen Toh, Steven James

**Affiliations:** 1 Faculty of Health Social Care and Medicine, Edge Hill University, St Helens Road, Ormskirk, L39 4QP, United Kingdom, 44 1695 575171; 2 Department of Paediatrics, Mersey and West Lancashire Teaching Hospitals, Ormskirk, United Kingdom; 3 CorrelAid e.V., Berlin, Germany; 4 Xibix Solutions GmbH, Unterschleißheim, Germany; 5 Action4Diabetes, Bangkok, Thailand; 6 Cambridge University, Cambridge, Cambridgeshire, United Kingdom; 7 University of the Sunshine Coast, Petrie, Queensland, Australia; 8 University of Melbourne, Parkville, Victoria, Australia; 9 Western Sydney University, Campbelltown, Victoria, Australia

**Keywords:** Action4Diabetes, diabetes, digital innovation, evidence, Southeast Asia

## Abstract

Recent decades have seen a dramatic proliferation of real-world data use and evidence generation from nonresearch settings. Data utilization is particularly revolutionizing the operations and impact of nongovernmental organizations worldwide, especially in low- and middle-income countries. Action4Diabetes, which has incrementally been providing sustainable diabetes care for children, adolescents, and young adults with type 1 diabetes (aged 0‐25 y) across Southeast Asia since 2015, is one such organization. Recognizing the importance of data, Action4Diabetes have collaborated with CorrelAid e.V. As part of this, Action4Diabetes has been exchanging patient data with the local program hospitals monthly. A preprocessing pipeline was implemented, extracting patient and medical product data in a standardized and unified manner. Data collected are anonymized and subsequently uploaded to secure public cloud storage, where they are processed and stored in a centralized database. The model used by Action4Diabetes shows that much can be achieved and can perhaps be utilized elsewhere.

## Introduction

Recent decades have seen a dramatic proliferation of the use of real-world data and evidence generation from nonresearch settings, including both health and nonhealth sources [1]. Data utilization is particularly revolutionizing the operations and impact of not-for-profit, nongovernmental organizations worldwide, especially in low- and middle-income countries where national health systems are limited [2]. This effort is vital for understanding health trends; identifying care and best practice gaps; supporting the design and evaluation of community-based sustainable, locally owned health solutions; and informing strategic planning and policy. Recognizing the importance of data, since February 2023, the nongovernmental organization Action4Diabetes [3-6] (Multimedia Appendix 1), which provides sustainable diabetes care to youth across Southeast Asia, has collaborated with CorrelAid e.V., a not-for-profit nongovernmental organization of data scientists [7]. This viewpoint aims to describe the preprocessing data pipeline that was implemented in collaboration, across Southeast Asia, to account for differences between files from different hospitals and over time for the same hospital; the pipeline was written in R and built on GitHub.

## Report

**Figure 1.**
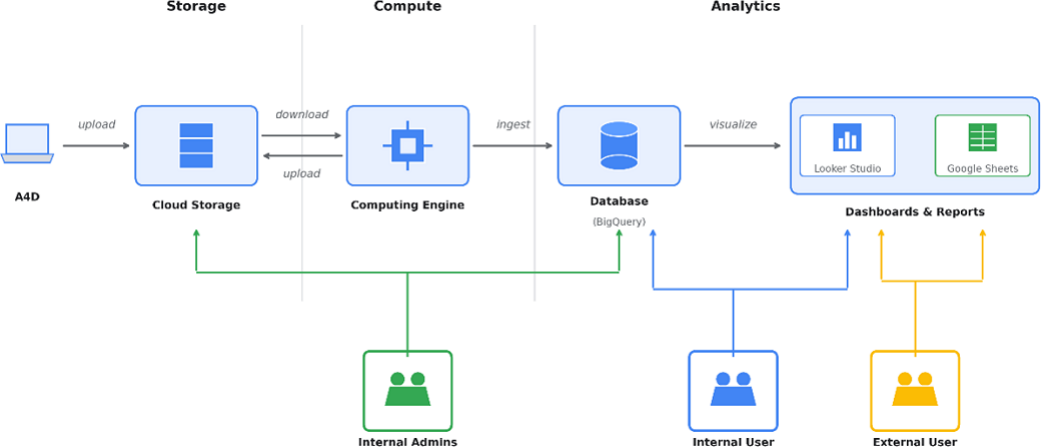
Data pipeline and cloud architecture with sample access patterns.

As part of the preprocessing data pipeline, Action4Diabetes has been exchanging patient data with local program hospitals monthly via Microsoft Excel (Microsoft Corp) files [8]. The pipeline extracts the patient and medical product data in a largely standardized and unified manner, rendering it suitable for database storage and enabling consistent regional analysis; privacy compliance is embedded through anonymization protocols prior to upload, mitigating legal and ethical risks, and automated preprocessing and error reporting and tracking mechanisms largely replace manual data cleaning, improving accuracy and reducing delays. Data collected are subsequently uploaded to secure storage in the public cloud, where it is processed and stored in a centralized database. Figure 1 illustrates the data pipeline and cloud architecture with sample access patterns.

We implemented the pipeline as an R package following a three-stage extract-clean-load architecture, with all source code version-controlled on GitHub. In the extraction stage, the pipeline reads monthly Excel worksheets from each hospital’s tracker file, identifies data boundaries using marker rows and header patterns, and maps column names to a canonical schema through a configuration-driven synonym file. This step is necessary because column naming conventions vary considerably across hospitals and have changed over the 2017‐2025 period; the synonym file currently maps over 60 patient variables and 14 product variables to their standardized equivalents.

The cleaning stage applies type conversions to every field and catches conversion errors through exception handling. Rather than silently dropping problematic records, the pipeline substitutes sentinel error values (eg, 999999 for numeric fields and 9999-12-31 for date fields) so that downstream analysts can trace exactly where data quality issues originate. Date parsing deserves particular mention because the trackers contain dates in many forms—Excel serial numbers, “15-Mar-2022,” “Mar 2022,” and occasionally dates embedded inside measurement strings—and the pipeline attempts each format in turn. For clinical measurements, the pipeline applies domain-specific rules: it strips inequality symbols from glycated hemoglobin values while retaining a threshold-exceeded flag, splits combined blood pressure strings into systolic and diastolic components, and converts height from centimeters to meters where the recorded value exceeds 50. Range validation then enforces clinically plausible bounds (eg, age 0‐100 years, glycated hemoglobin 0%‐25%) and replaces outliers with error sentinels. Categorical fields such as insulin type and patient status pass through allowed-value checks defined in a YAML configuration file, and the pipeline logs every validation event as structured JSON with a standardized warning code, which makes it straightforward to audit data quality across sites and reporting periods. In the final stage, the pipeline assembles cleaned records into database-ready tables, selecting the relevant columns and ordering by patient and date for ingestion.

Anonymization takes place before any of the above processing begins. A separate Python tool reads the “Patient List” sheet from each tracker to build a lookup between patient names and unique program identifiers, which follow a structured format: a two-letter country code, a two-letter clinic code, and a three-digit sequential number. The tool then walks through every worksheet in the workbook and replaces each name with its corresponding identifier, saving the deidentified copy to a separate directory. Original files never leave secure local storage. During pipeline processing, the R code validates each identifier against the expected pattern and logs any mismatches for manual review. Once cleaning is complete, the pipeline serializes data to Apache Parquet and uploads it to Google Cloud Storage, from where it feeds into Google BigQuery—a managed columnar data warehouse that serves as the single point of access for analysis. Access control follows Google Cloud Identity and Access Management, with role-based permissions for internal administrators, standard users, and external collaborators (Figure 1); all storage buckets are private and restricted to authenticated project members.

The process of developing a data pipeline that ingests historical data and makes it accessible in a centralized database in a single place for further analysis took approximately 1 year to complete. The subsequent process stages entailed the identification of initial use cases that build upon the data, the implementation of dashboards and reports that facilitate data access, the automation of the data pipeline to ingest newly arriving data without manual intervention, and finally the establishment of a data governance strategy to oversee data content, structure, usage, and safety. Currently, the cloud database provides access to all historical data for the years 2017‐2025 inclusive, encompasses 7 countries, 42 clinics, and approximately 1400 patients. Furthermore, the database contains information about the distribution and usage of approximately 200 medical products.

The impact of this work has been well documented. For example, as of August 16, 2021, 45 children, adolescents, and young adults with type 1 diabetes who were enrolled in Action4Diabetes’s clinic support program constituted the first known cohort of Laotians to have survived a diagnosis of type 1 diabetes [5]. Given that as recently as 2016, no child was known to have lived with type 1 diabetes into adulthood in the Lao People’s Democratic Republic, this is indeed a remarkable achievement for a lower-middle income country in Southeast Asia.

Despite its innovations, however, the Action4Diabetes pipeline faces several challenges that impact program efficiency and evaluation. Such challenges include fragmented data sources, creating inconsistencies and requiring extensive manual harmonization, and limited automation in data validation, meaning some errors still require manual correction. Further, the pipeline depends on Excel-based inputs, which restricts real-time integration and increases formatting inconsistency risk, while infrastructure gaps in partner hospitals—such as reliance on paper records—limit data completeness and accuracy, a problem also evident in high-income countries [9]. Finally, predictive analytics capabilities are basic, focusing mainly on supply forecasting rather than advanced patient risk stratification, and governance and interoperability challenges persist across multiple countries, with varying regulations and data-sharing agreements hindering streamlined data sharing and governance. These limitations highlight the need for further investment in automation, mobile data capture, and regional governance frameworks.

To enhance the effectiveness and sustainability of the Action4Diabetes data pipeline, several improvements are recommended. Transitioning from manual file uploads to secure application programming interface–based data exchange would reduce errors and improve timeliness. Expanding real-time dashboards for hospitals and health authorities could enable dynamic monitoring of patient trends and insulin stock levels. Integrating mobile data capture tools for frontline staff would minimize reliance on paper records and improve data completeness. Additionally, incorporating advanced predictive analytics such as machine learning models could support demand forecasting and early identification of high-risk patients [10]. Finally, formalizing regional governance frameworks and data-sharing agreements would ensure compliance, interoperability, and streamlined collaboration across multiple countries.

## Limitations

The data pipeline and resulting dataset have several limitations that users of the data should bear in mind. Because the Excel tracker templates have evolved over the period 2017‐2025—for instance, individual insulin type checkboxes only appeared from 2024 onward—earlier records systematically lack variables that are present in more recent data. This creates a temporal bias: longitudinal analyses will inevitably draw on richer information for recent cohorts than for historical ones. Data completeness also varies across hospitals, largely as a function of local clinical capacity and staff familiarity with the tracker. In practice, this means that facilities with more established data entry routines contribute more complete records, and any analysis requiring complete cases will skew toward these better-resourced sites and the populations they serve.

Data fragmentation compounds the problem. Each hospital maintains its own tracker with locally adapted column names and entry conventions. The synonym-based harmonization catches most of these variations, but any field that does not match a known synonym is quietly excluded, so site-specific clinical observations can be lost. Manual data entry by clinicians—who understandably prioritize patient care over spreadsheet accuracy—introduces missing values, typographical errors, and inconsistent use of categorical codes (eg, free-text insulin regimen descriptions rather than the standardized categories the pipeline expects). The validation framework flags and logs these issues rather than silently discarding data, but the sentinel error values it substitutes must be handled carefully in any downstream analysis to avoid distorting summary statistics.

There is also a question of representativeness. The dataset captures only those patients enrolled in the Action4Diabetes program at participating hospitals; individuals with type 1 diabetes who are managed elsewhere or who have not been enrolled do not appear. In countries where program coverage is still limited, the data therefore cannot speak for the broader type 1 diabetes population. The monthly reporting cadence introduces a further constraint: clinical events that occur between reporting windows may be recorded with reduced temporal precision or missed altogether if not entered retrospectively. Finally, the anonymization approach—a deterministic name-to-identifier substitution—depends on the accuracy of each hospital’s “Patient List” reference sheet. If a name is misspelled or absent from that list, the corresponding records will not be fully deidentified; the tool logs these cases for manual follow-up, but they represent a residual privacy risk that we actively monitor. Despite these constraints, the pipeline offers a pragmatic and reproducible way to harmonize messy real-world clinical data across a multicountry program, and its structured logging makes the boundaries of data quality transparent to anyone working with the resulting dataset.

## Conclusion

The implementation of a centralized database solution for diabetes-related health care data is a challenging but also achievable objective when approached in a cost-effective manner, particularly when using services from modern cloud providers. With the current diabetes epidemic and projections for future decades illustrating a worsening state [11], the significance of data will likely continue to expand, enabling nongovernmental organizations to more effectively address the needs of communities they serve. The model used by the nongovernmental organization Action4Diabetes across Southeast Asia shows that much can be achieved and can perhaps be utilized elsewhere.

## Supplementary material

10.2196/89357 Multimedia Appendix 1 Action4Diabetes.

## References

[1] Gregg EW, Patorno E, Karter AJ (2023). Use of real-world data in population science to improve the prevention and care of diabetes-related outcomes. Diabetes Care.

[2] Nikita NA, Azim KS, Jafor AHM, Shayed AU, Hossain MA, Khan OU (2024). Digital transformation in non-profit organizations: strategies, challenges, and successes. AIJMR.

[3] Toomey C, Gore J, Ooi F (2021). Action4Diabetes: a UK charity revolutionising type 1 diabetes healthcare across South-East Asia. Diabetes Care for Children and Young People.

[4] Ng SM (2023). Action4Diabetes: a non‐profit organisation bridging the type 1 diabetes gap in Southeast Asia. Practical Diabetes.

[5] Lek N, Manivong A, Rassavong K, Phommachack D, Toomey C, Ng SM (2022). Type 1 diabetes in Laos, 2016-2021. Pediatr Diabetes.

[6] Ng SM, Malene IV, Nguyen TK (2023). Internet analytics of an innovative digital educational resource of type 1 diabetes HelloType1 in local languages for people living with diabetes families and healthcare professionals in Southeast Asia. BMC Endocr Disord.

[7] Wir bringen daten in die zivilgesellschaft!. CorrelAid ev.

[8] Ng SM, Malene IV, Nilar M (2022). Closing the Type 1 Diabetes gap in South-East Asia through government partnership working with non-government organisations. Diabetes Res Clin Pract.

[9] Best J (2023). NHS still reliant on paper patient notes and drug charts despite electronic upgrades, *The BMJ* finds. BMJ.

[10] Lam JYJ, Barras M, Scott IA, Masood H, Abdel-Hafez A, Falconer N (2026). Machine learning risk prediction models for medication harm in hospitalised adult patients. Ther Adv Drug Saf.

[11] IDF diabetes atlas 2025. https://diabetesatlas.org/resources/idf-diabetes-atlas-2025.

