# Supplementary material for: Harnessing Digital Innovation for Diabetes Care: Insights From the Action4Diabetes–CorrelAid Data4Good Collaboration
Source: JMIR Diabetes. 2026 Apr 17;11:e89357. doi: 10.2196/89357 (PMC13089624; doi:10.2196/89357)
Supplement: Multimedia Appendix 1 [file diabetes-v11-e89357-s001.docx]

**Supplementary material 1: Action4Diabetes**

Action4Diabetes, registered in the United Kingdom and Thailand, has been providing sustainable diabetes care for children, adolescents and young adults with type 1 diabetes (aged 0-25 years) in Myanmar, Thailand, the Lao People’s Democratic Republic (since 2015-2016), and Cambodia, Vietnam and Malaysia (since 2017-2019) [3, 4, 8], and is currently expanding its reach to Indonesia and the Philippines. Such care is provided in partnership with defined local hospitals, with memorandum of understandings signed with applicable governments guaranteeing ongoing supplies of free insulin, blood glucose testing kits and hospital emergency funds. Besides health capacity building, Action4Diabetes has four key programs, each of which work together to guarantee the support of a child, adolescent and young adult with type 1 diabetes: 1.) life-saving supplies like insulin and monitoring equipment; 2.) education and empowerment through online resources and training; 3.) capacity building for healthcare professionals; and 4.) advocacy for care access by partnering with local and international organisations .
